# Isocyanides: Promising Functionalities in Bioorthogonal Labeling of Biomolecules

**DOI:** 10.3389/fchem.2021.670751

**Published:** 2021-04-29

**Authors:** Yuchen Zhu, Jia-Yu Liao, Linghui Qian

**Affiliations:** ^1^ College of Pharmaceutical Sciences, Zhejiang University, Hangzhou, China; ^2^ Hangzhou Institute of Innovative Medicine, Zhejiang University, Hangzhou, China; ^3^ Cancer Center, Zhejiang University, Hangzhou, China; ^4^ Zhejiang Province Key Laboratory of Anti-Cancer Drug Research, College of Pharmaceutical Sciences, Zhejiang University, Hangzhou, China

**Keywords:** bioorthogonal chemistry, biomolecule labeling, isocyanide, ligation, cleavage

## Abstract

Isocyanides have drawn increasing attention in biological applications due to their attractive properties and unique reactivities, which can undergo various reactions, such as multicomponent reactions, α-addition reactions, [4 + 1] cycloaddition reactions, and the reaction scope keeps expanding. In addition to acting as reactants for the preparation of structurally interesting and diverse *N*-heterocycles or peptidomimetics, this type of functionality may be a good choice in the labeling and modulation of biomolecules due to the high biocompatibility and small size to minimize modifications on the parent molecule. It has been demonstrated that isocyanides can participate in biomolecule labeling through three strategies, including the two-component bioorthogonal reaction, multicomponent reaction, and metal chelation. Among them, the isocyanide-tetrazine reaction has been better studied recently, augmenting the potency of isocyanide as a bioorthogonal handle. This review will focus on the recent progress in isocyanide chemistry for labeling of biomolecules. Meanwhile, methods to introduce isocyano groups into biomacromolecules are also described to facilitate wider applications of this unique functionality.

## Introduction

**FIGURE 1:**
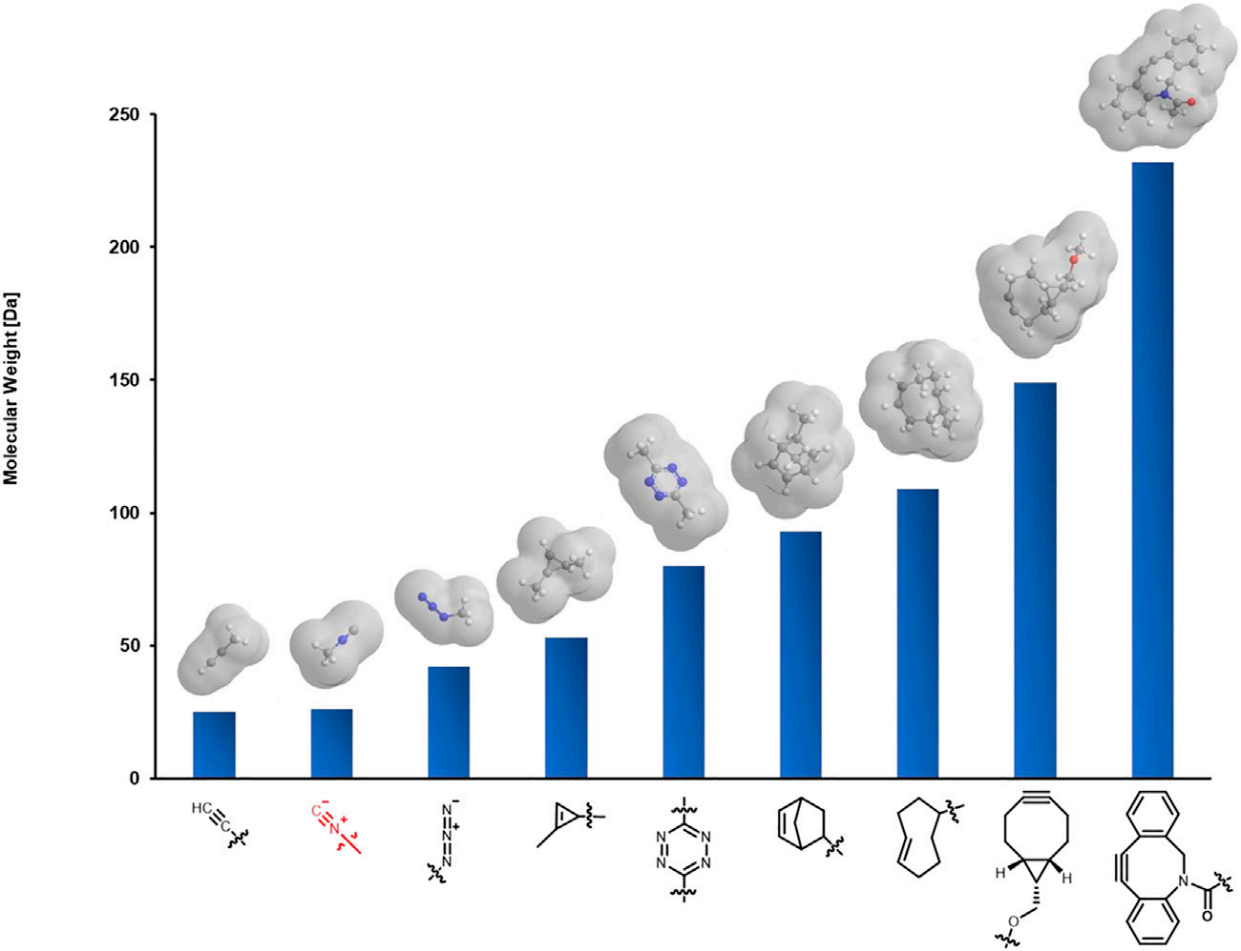
Comparison of the size of isocyano group with other commonly used bioorthogonal functionalities. Molecules are shown with the corresponding methyl derivatives.

Isocyanides are a class of highly versatile and irreplaceable reagents as the isocyano group may serve as both nucleophile and electrophile in chemical reactions (Nenajdenko, 2012). Over the past several decades, isocyanide chemistry has drawn much attention and found wide applications in synthetic organic and medicinal chemistry for the construction of structurally interesting and diverse *N*-heterocycles or peptidomimetics. The classical reactions involving isocyanides include but not limited to multicomponent reactions (MCRs), such as the well-known Passerini and Ugi reaction, α-addition reactions with electrophilic substrates, as well as [4 + 1] cycloaddition reactions. Since many of the above reactions could take place smoothly in aqueous media, and isocyanides are stable at biologically relevant pH [stable at pH 4–9 (Goldstein and Niv, 1993)] without obvious biotoxicity (Deb and Franzini, 2020), isocyanide chemistry has been successfully employed to realize the labeling of biomacromolecules, including proteins and polysaccharides. More intriguing, the isocyano group with a bond length of only 1.17 Å is known to date as the smallest bioorthogonal functionality suitable for living systems (Figure 1; though terminal alkyne is a bit smaller in size, it cannot be readily used in living systems through Cu-catalyzed azide-alkyne cycloaddition), allowing to modify the target biomolecule with minimum interference in the structure and function, thus may be suitable for cellular, subcellular, and even *in vivo* imaging. Other than bioimaging, it is also expected to achieve the activation of specific proteases and targeted drug release when used in combination with other strategies (Zhang et al., 2020). To facilitate wider applications of isocyanides in biological labeling, three strategies developed recently will be systematically described here.

## Strategy 1: Isocyanide as a Bioorthogonal Handle

Bioorthogonal reactions are chemical reactions that neither interact nor interfere with a biological system. They have the following properties: 1) rapid reaction rate at low concentrations; 2) chemical selectivity, free from the influence of electrophilic and nucleophilic residues in biomolecules; 3) both reactants and products are stable under physiological conditions; 4) the products are small and chemically inert to avoid interfering with the activity of the target biomolecule (Kenry and Liu, 2019). Traditionally, bioorthogonal chemistry has largely been viewed as two-component “ligation” reactions since Bertozzi et al. proposed the term of bioorthogonal reaction in 2003 (Lemieux et al., 2003; Prescher and Bertozzi, 2005) to describe the biocompatible Staudinger ligation between an azido-containing unnatural sugar displayed on the living cell surface and a modified triphenylphosphine-fluorophore conjugate. To date, there are many bioorthogonal reactions available, such as Cu-catalyzed azide-alkyne cycloaddition (CuAAC), strain-promoted (Cu-free) azide-alkyne cycloaddition (SPAAC), inverse electron demand Diels-Alder (IEDDA) reactions including the tetrazine ligation and the isocyanide-based click reaction (Patterson et al., 2014; Smeenk et al., 2021). These reactions have been widely used in the labeling of biomolecules. In 2013, Robillard et al. reported the “click to release” reaction between tetrazine and *trans*-cyclooctene (TCO, with a carbamate group next to the double bond), initiating the release of drugs from the TCO (Versteegen et al., 2013). Such bioorthogonal bond cleavage reactions were thoroughly reviewed by Chen’s group in 2016, highlighting their applications in regulating cellular proteins or manipulating biological functions (Li and Chen, 2016; Zhang et al., 2016). Both the bioorthogonal ligation and cleavage reactions have expanded the chemical toolkit for biological discovery, and functionalities such as isocyanides that can undergo either ligation or cleavage controlled by their substituents will be particularly useful.

### Isocyanide-Tetrazine Chemistry

**FIGURE 2:**
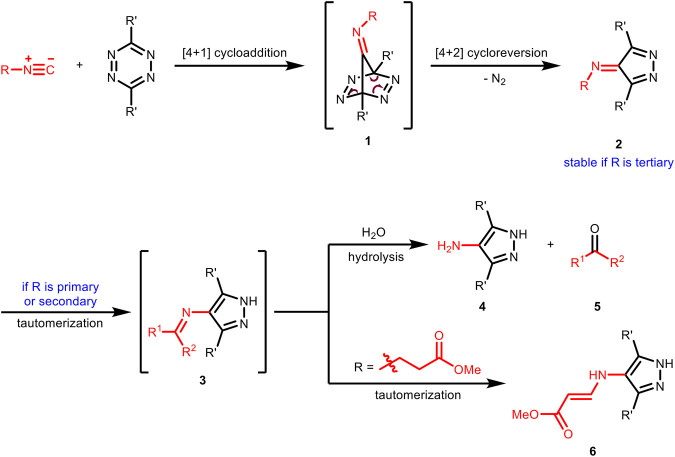
Mechanism of the reaction between isocyanides and tetrazines.

As early as 1982, Seitz et al. reported the first example of [4 + 1] cycloaddition reaction of benzyl isocyanides with tetrazines to prepare pharmacologically interesting amino-pyrazoles (Imming et al., 1982). The first step of this transformation is the formation of tetraazanorbornadienimine derivatives **1** through [4 + 1] cycloaddition. This type of intermediate was unstable and would spontaneously undergo [4 + 2] cycloreversion with the release of N_2_ to result in imine derivatives **2**, which would further tautomerize to the aromatic pyrazoles **3** and then hydrolyze to amino-pyrazoles **4** and carbonyls **5** (Figure 2). Because of the stability issue of **2**, this [4 + 1] cycloaddition reaction could not be used directly for chemical ligation reactions in aqueous media. In 2011, Leeper et al. investigated this chemistry systematically with different types of isocyanides (Stöckmann et al., 2011). They found that for primary and secondary isocyanides, the imines **2** could not be isolated and the corresponding amino-pyrazoles and aldehydes/ketones were obtained as the final products in the same manner with Seitz’s report. In contrast, the imine products **2** from the reaction of tertiary isocyanides was found to be stable as the tautomerization process (from **2** to **3**) was prohibited. Besides, imines **3** formed from propanoate-derived isocyanides did not hydrolyze either, but instead tautomerized again to give the vinylogous urethane derivatives **6** (Figure 2).

**FIGURE 3:**
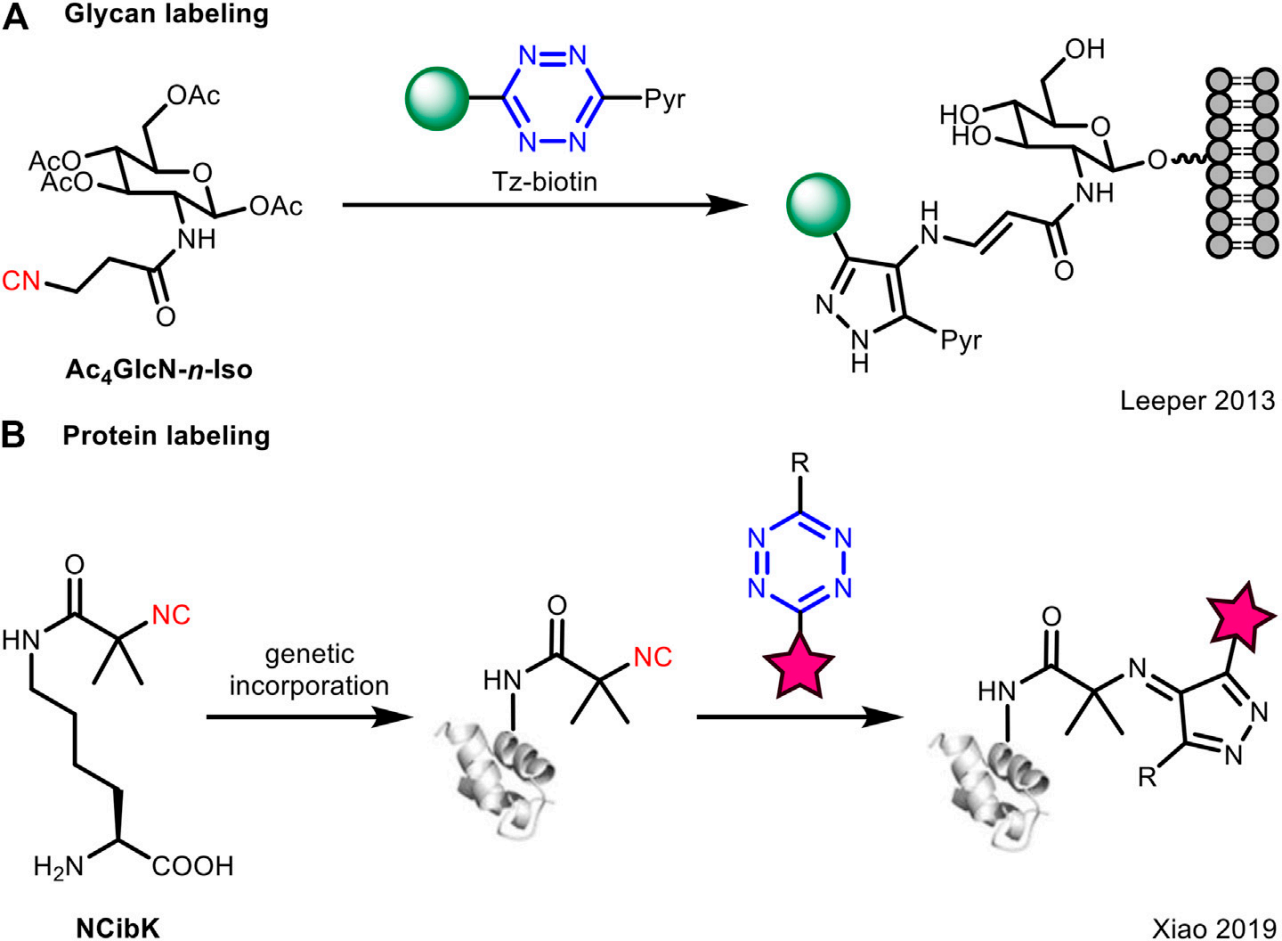
Application of isocyanide-tetrazine chemistry for bioorthogonal ligation with **(A)** glycans or **(B)** proteins.

It is noteworthy that the isocyanide-tetrazine [4 + 1] cycloaddition is much slower than the conventional counterpart of tetrazine such as TCO, and this may limit its application in biomolecule labeling (Stairs et al., 2013). To compensate for the low reaction rate, Leeper et al. employed a high concentration of Tz-biotin (non-fluorogenic) for conjugation to primary 3-isocyanopropanamide-derived glycans (**Ac**_**4**_ **GlcN-*n*-Iso**) on the cell surface (Figure 3A). However, this method did not essentially solve the kinetic problem between isocyanides and tetrazines. Later, Xiao et al. (Chen et al., 2019) designed a *tert*-butyl isocyano group conjugated lysine (**NCibK**) for protein labeling via [4 + 1] cycloaddition reaction with tetrazine probes (Figure 3B). The unnatural amino acid **NCibK** was introduced into proteins via genetic code expansion technology. This was the first example to use isocyanide-tetrazine ligation to achieve protein labeling, though the reaction rate is not high.

**FIGURE 4:**
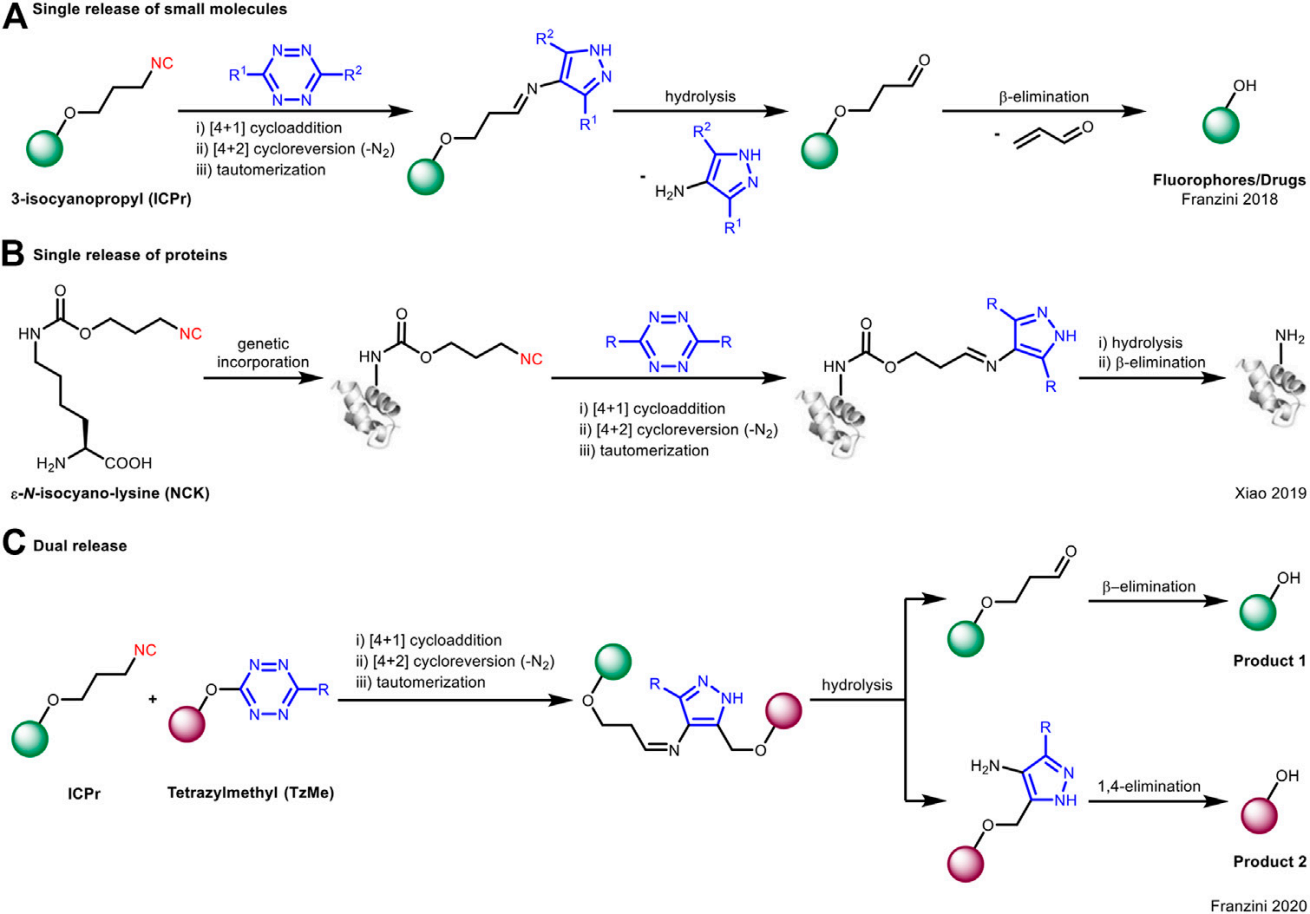
Application of isocyanide-tetrazine chemistry for bioorthogonal cleavage in **(A)** single release of small molecules, **(B)** single release of proteins, or **(C)** dual release of fluorophores.

On the contrary, Franzini et al. took advantage of the spontaneous hydrolysis of the cycloaddition product from primary isocyanide and tetrazine for “click to release” (Tu et al., 2018). In this case, they used 3-isocyanopropyl (**ICPr**) substituents as masking groups which can be effectively removed upon reacting with tetrazines to liberate phenol-containing active molecules under physiological conditions (Figure 4A). This concept could be extended to the release of proteins (Figure 4B; Chen et al., 2019). Furthermore, by the use of both primary isocyanide and tetrazine as “protecting groups,” the caged fluorophores would undergo bioorthogonal bond cleavage to liberate dual fluorophores through a single reaction (Figure 4C; Tu et al., 2020).

Meanwhile, Franzini’s group thoroughly explored the structure-activity relationship of the reaction between isocyanides and tetrazines (Table 1), and found that tetrazines with bulky substituents could form stable adducts with primary isocyanides, thus obviating the need for engineering the isocyanide moiety (e.g., tertiary isocyanides) (Tu et al., 2019; Xu et al., 2019). What’s more interesting is that stable asymmetric tetrazines with bulky and electron-withdrawing substituents (**Tz-4**) can react with isocyanides rapidly under high-water conditions (Table 1, Entries 12–13). Computational analysis showed that dispersion forces between the isocyano group and the bulky tetrazine substituents in the transition state contribute to the acceleration. The use of sterically hindered tetrazine allows the rapid conjugation with primary/secondary isocyanides, thus promoting isocyanides to be among the most promising bioorthogonal reporters.

**TABLE 1:** Second-order rate constants of reactions between tetrazines and different counterparts (structures of these compounds are shown in Supplementary Material).

Entry	Tetrazine	Counterpart	*k*_*2*_ [M^−1^S^−1^]	Condition
1	Tz-1	*t*-OcNC	1.48 ± 0.03	DMSO/H_2_O = 4:1, *T* = 37°C
2	Tz-1	Cp	0.563 ± 0.025	DMSO/H_2_O = 4:1, *T* = 37°C
3	Tz-1	Nb	0.827 ± 0.042	DMSO/H_2_O = 4:1, *T* = 37°C
4	Tz-1	TCO	>100	DMSO/H_2_O = 4:1, *T* = 37°C
5	Tz-2	PhEtNC	1.15 ± 0.20	DMSO/H_2_O = 4:1, *T* = 37°C
6	Tz-3	PhEtNC	1.53 ± 0.01	DMSO/H_2_O = 4:1, *T* = 37°C
7	Tz-4	PhEtNC	2.42 ± 0.10	DMSO/H_2_O = 4:1, *T* = 37°C
8	Tz-4	*t*-OcNC	6.27 ± 0.05	DMSO/H_2_O = 4:1, *T* = 37°C
9	Tz-4	Cp	0.588 ± 0.055	DMSO/H_2_O = 4:1, *T* = 37°C
10	Tz-4	Nb	0.013 ± 0.005	DMSO/H_2_O = 4:1, *T* = 37°C
11	Tz-4	TCO	0.234 ± 0.002	DMSO/H_2_O = 4:1, *T* = 37 °C
12	Tz-4	PhEtNC	29.4 ± 0.8	DMSO/H_2_O = 1:4, *T* = 37°C
13	Tz-4	*t*-OcNC	57 ± 5	DMSO/H_2_O = 1:4, *T* = 37°C

Data sources: Tu et al., 2019.

### Isocyanide-Chlorooxime Chemistry

**FIGURE 5:**
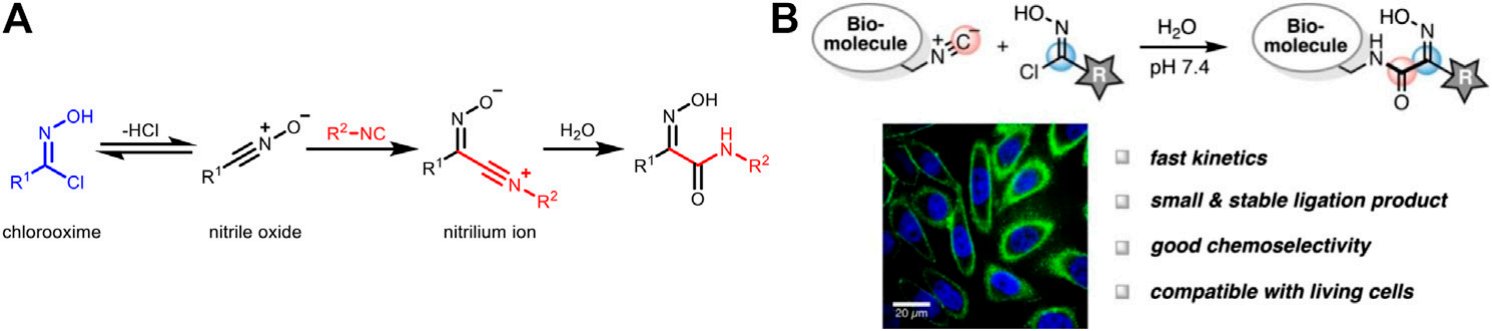
**(A)** Mechanism of the reaction between isocyanides and chlorooximes. **(B)** Isocyanide-chlorooxime ligation for the labeling of biomolecules in live cells. Re-printed with permission from American Chemistry Society Publications 2019 (Schäfer et al., 2019).

Other than tetrazines, Helma et al. successfully applied the classical reaction of isocyanides with chlorooximes (Figure 5A) for bioorthogonal ligation (Figure 5B; Schäfer et al., 2019). It showed a modest reaction rate (*k*_2_ ≈ 1 M^−1^ s^−1^) with conversion greater than 95%. Moreover, this reaction is highly selective, thus compatible with other bioorthogonal reactions when used in combination. In this study, isocyanide-modified *N*-acetylmannosamine derivatives (carbohydrate metabolism precursor) was incubated with live cells, and then a biotin probe containing a chlorooxime group was added. There was a significantly enhanced fluorescence signal after incubation, highlighting the capability of isocyanide-chlorooxime ligation for live-cell labeling.

## Strategy 2: Isocyanide-based MCR

**FIGURE 6:**
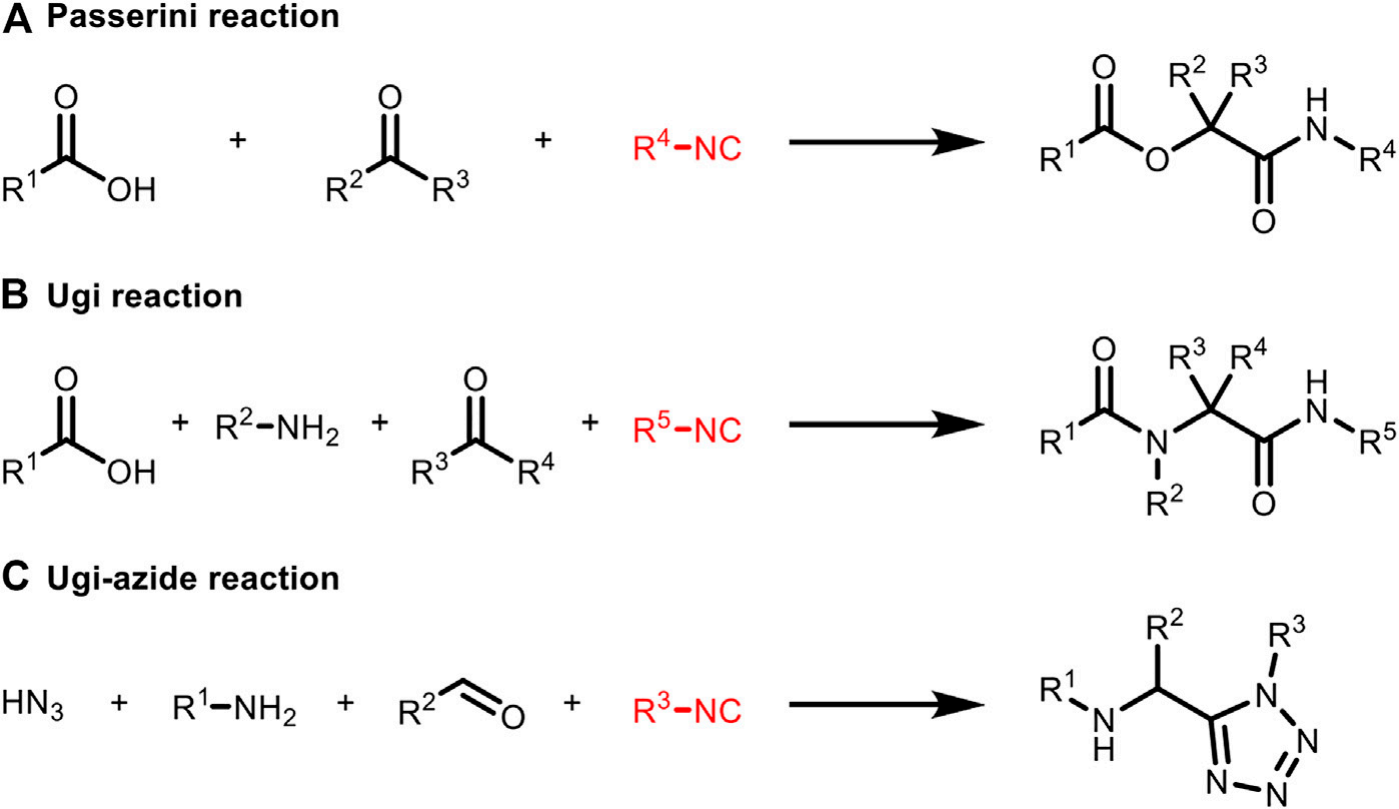
Isocyanide-based MCRs applied to bioconjugation include **(A)** Passerini reaction, **(B)** Ugi reaction and **(C)** Ugi-azide reaction. All the substituents (R^1^−R^5^) can be biomolecular fragments.

In 1983, Vodrážka et al. applied Ugi reaction to immobilize glucose oxidase on a polymer carrier which was the earliest report of protein bioconjugation based on MCR (Marek et al., 1983). In 2000, Lang et al. reported that the Ugi reaction was used to achieve the binding of bovine serum albumin (BSA) with isocyano-modified β-D-glucosyl groups (Ziegler et al., 2000). This is the first proof that isocyanide can undergo a Ugi reaction under physiological conditions. Recently, Rivera et al. gave a detailed description of the multicomponent coupling of various biomacromolecules, including those employing isocyanides (Figure 6; Reguera et al., 2018).

**FIGURE 7:**
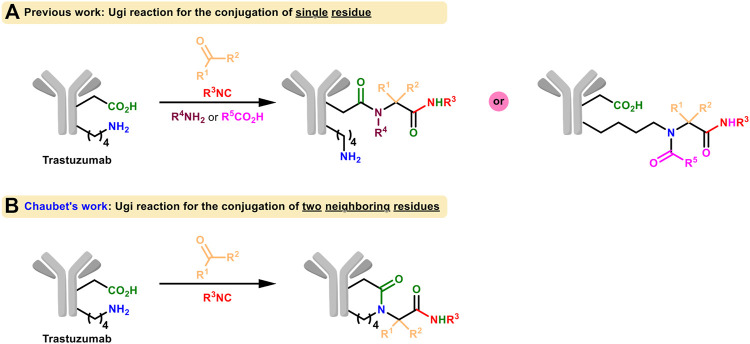
**(A)** Ugi reaction for the conjugation of single residue of native proteins. **(B)** Ugi reaction for the conjugation of two neighboring residues of native proteins.

More examples using isocyanide as one of the MCR components for protein labeling were presented afterwards (Figure 7A). Very recently, the side-chain amine and carboxylic groups of two neighboring lysines and aspartate/glutamate on trastuzumab were conjugated in the presence of isocyanide and aldehyde through Ugi reaction by Chaubet et al. to achieve site-selective modification (Figure 7B; Sornay et al., 2020). This method can be used to obtain antibody conjugates with different payloads as well as to label native proteins in a site-specific manner. But due to the requirement of multicomponent coordination, it is not practical for living systems yet.

## Strategy 3: Isocyanide as a Coordination Ligand for Transition Metals

**FIGURE 8:**
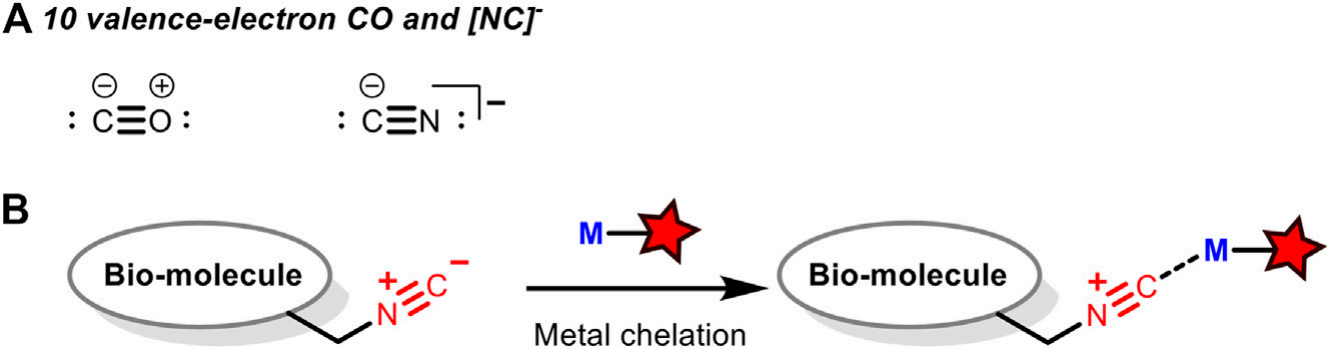
**(A)** 10 valence-electron CO and [NC]^−^ that function as terminal ligands to transition metals. **(B)** Isocyano-containing biomolecules can be labelled via metal chelation. M = transition metal.

Akin to carbon monoxide as a strong σ-donor and a good π-acceptor, isocyanides could coordinate with transition metals. Therefore, it is possible to design transition metal-based fluorescent probes to recognize biological macromolecules tagged with isocyanides through their coordination. Although there is no report on such applications yet, there are many related researches to detect carbon monoxides with transition metal-derived fluorescent probes (Alcarazo et al., 2009; Yuan et al., 2013; Kos and Plenio, 2015; Alday et al., 2020). As isoelectronic with carbon monoxide (Figure 8A), isocyanide may also be used as a ligand which can be recognized by such fluorescent probes to achieve the non-covalent fluorescent labeling of biomacromolecules (Figure 8B).

## Methods to Introduce the Isocyano Group Into Biomacromolecules

**FIGURE 9:**
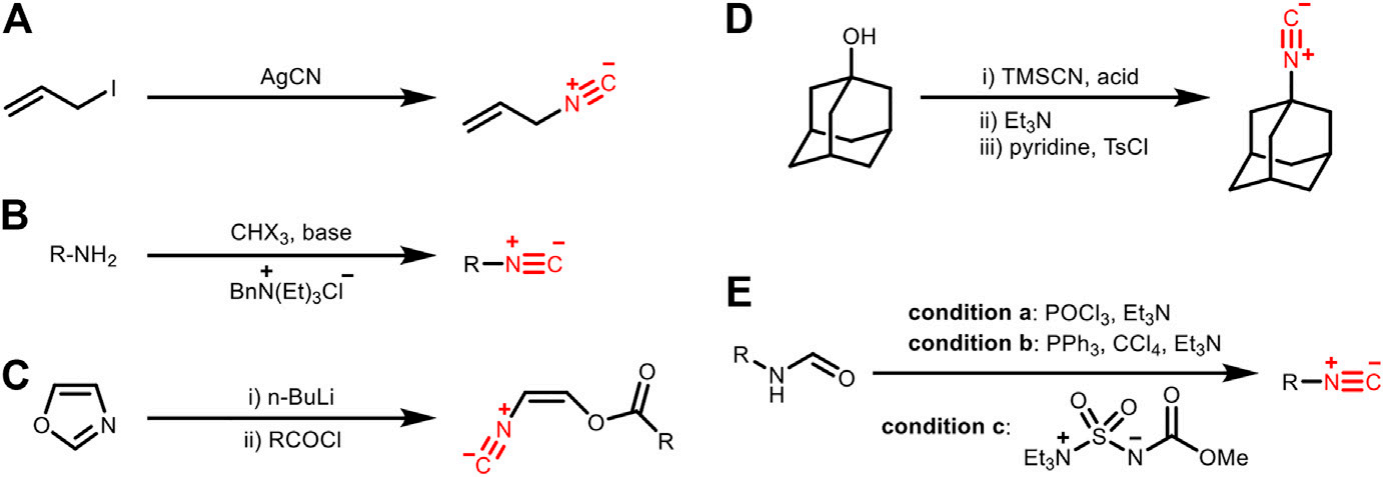
Preparation of isocyanides by **(A)** the reaction of silver cyanide with alkyl iodide, **(B)** Hoffman carbylamine reaction with phase transfer catalysis, **(C)** ring-opening reaction of oxazole, **(D)** one-pot reaction using trimethylsilyl cyanide (TMSCN) and benzylic/tertiary alcohol, or **(E)** dehydration of formamide.

There are many methods for introducing the isocyano group into small molecules, among which five of them are classic (Figure 9): A) by the reaction of silver cyanide with alkyl iodide; B) Hoffman carbylamine reaction with phase transfer catalysis; C) ring-opening reaction of oxazole to prepare unsaturated isocyanides; D) one-pot reaction using trimethylsilyl cyanide (TMSCN) and benzylic or tertiary alcohol; E) dehydration of formamide (Bode et al., 2016). Among these methods, the dehydration of formamide is the most popular one. In contrast, how to introduce the isocyano group into biomacromolecules like proteins and carbohydrates for subsequent bioorthogonal modification (i.e., labeling or modulation through bond cleavage) has been less addressed.

### Introducing the Isocyano Group Into Proteins

#### With Bifunctional Linkers

**FIGURE 10:**
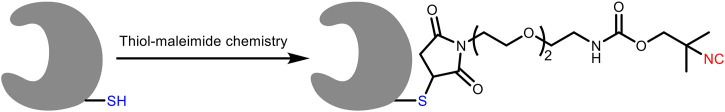
An isocyano-containing linker tagged to the protein through cysteine ligation.

In 2011, Leeper et al. labeled the cysteine residues on the protein with the isocyano group via an isocyano-maleimide bifunctional linker (Figure 10; Stöckmann et al., 2011). However, a problem with this strategy is that the C-S bond is not stable under physiological conditions, and reverse Michael addition may cause cleavage of the isocyanide side chain. With efforts made in recent years to improve the stability of the Michael addition adduct, this problem can be properly addressed (Huang et al., 2019).

#### By Site-Directed Insertion of Unnatural Amino Acids

This strategy is among the mainstream for protein modification. The unnatural amino acid containing an isocyano group can be inserted into the protein by two methods, namely the selective pressure incorporation (SPI) and the stop codon suppression (SCS) (Drienovská and Roelfes, 2020). The SPI method is based on that some tRNAs can recognize and bind those unnatural amino acids with structures similar to natural amino acids. When there are not enough natural amino acids in the biological system (auxotrophic), the insertion of unnatural amino acids can be realized. However, this method highly relies on the structure of unnatural amino acids and may randomly insert unnatural amino acids into different sites of the protein, which may affect its structure and function. The SCS method is more widely used, which introduces an exogenous translation system (including tRNA and aminoacyl tRNA synthetase) that does not recognize endogenous amino acids but can specifically recognize unnatural amino acids (Fu et al., 2018). At present, more than 200 unnatural amino acids can be inserted into protein in this way, including the isocyanide derivatives.

#### Introducing the Isocyano Group Into Carbohydrates

**FIGURE 11:**
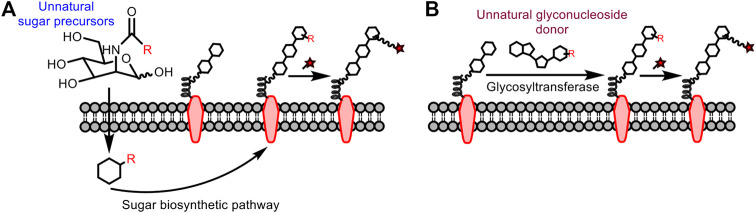
**(A)** Metabolic oligosaccharide engineering. **(B)** Chemoenzymatic glycan labeling.

Traditional glycan analysis methods include chemical derivatization (e.g., film method, dinitrosalicylic acid method), pre-column derivatization (e.g., sugar chains with fluorescent groups to be analyzed by HPLC, etc., ), and biological macromolecule recognition (such as antibodies and clusterins) (Ghazarian et al., 2011). However, the chemical derivatization is not selective; the pre-column derivatization is destructive to sugars and not practical for *in vivo* applications; the biological macromolecule recognition is faced with the problem of low affinity for sugar. To this end, methods suitable for analyzing polysaccharides in living systems are in high demand. To insert the bioorthogonal handle into the sugar for subsequent fluorescent modification, there are mainly two strategies (Figure 11), metabolic oligosaccharide engineering (Dube and Bertozzi, 2003) and chemoenzymatic glycan labeling (Lopez Aguilar et al., 2017).

#### Metabolic Oligosaccharide Engineering

This strategy was first proposed by Reutter’s group (Kayser et al., 1992). They found that unnatural analogues of mannosamine carrying a functional reporter can be used as metabolic precursors to participate in the biosynthetic pathway of sialic acid *in vivo*, after which the mannosamine can be visualized following conjugation with the fluorophore. In addition, isocyanide-based bioorthogonal reactions can be carried out smoothly even with the endogenous glycans in low abundance. However, the reporter must be small enough to be tolerated by the sugar biosynthetic system. In 2013, Leeper et al. tried to label glycans through sugar metabolism of *N*-acetyl-glucosamine and mannosamine derivatives containing isocyano groups, and then to use fluorophores with tetrazine for conjugation (Stairs et al., 2013). In 2019, Wennemers et al. also used this idea to incubate isocyano-modified *N*-acetylmannosamines with cells followed by the addition of biotin probes containing chlorooxime (Schäfer et al., 2019). Both studies demonstrate the potential of isocyanide as a bioorthogonal reporter for glycan labeling. However, it still has limitations: the sugar precursor may participate in multiple metabolic pathways after entering the cell to form various tagged sugars, thus not applicable for specific labeling.

#### Chemoenzymatic Method

As mentioned above, metabolic engineering through monosaccharide cannot meet the requirement to label more complex sugar chains. The chemoenzymatic method transfers monosaccharide analogues in the form of nucleotide sugar donors to specific glycan acceptors through glycosyltransferase, so that the glycan acceptors are equipped with the functional tag from the donor. This method was first reported by Hill et al. (Paulson et al., 1979) and the bioorthogonal reaction involving isocyanides is expected to be compatible with more complicated sugars in addition to monosaccharides. To be noted, this chemoenzymatic method highly relies on the development of glycosyltransferases that are tolerable to unnatural sugar donors and specific to the glycan acceptor.

#### Introducing the Isocyano Group Into DNA

Other than the important biological roles, DNA can also work as versatile tags for organic compounds. As early as 1992, Brenner and Lerner proposed the concept of adding a DNA barcode to chemically synthesized molecules to form DNA-encoded libraries (Brenner and Lerner, 1992). MCRs proved to be compatible with solid-phase synthesis and can be effective for creating scaffold diversity with a low synthetic cost (Rivera et al., 2021). In 2019, Brunschweiger’s team reported isocyanide-involved MCRs for DNA-encoded combinatorial synthesis (Kunig et al., 2019). This concept has great potential to be applied for fluorescence labeling or other derivatizations of DNA with isocyanides as well as to introduce an isocyano group into DNA.

## Perspectives

The isocyano functionality has many advantages such as small size, unique reactivity, as well as good biocompatibility, thus are considered as promising bioorthogonal handles. To undergo a bioorthogonal reaction, there should be a suitable counterpart allowing good reaction kinetics and ideal products. Therefore, the application of isocyanides in biological systems increased rapidly after the finding that large sterically hindered tetrazines can form stable products rapidly after conjugation with primary isocyanides. Very recently, chlorooxime is found to be another complementary counterpart for bioorthogonal ligation with isocyanides, further expanding the bioorthogonal chemistry repertoire involving isocyanides.

Previously, Shasqi et al. employed the tetrazine-TCO reaction to achieve the enrichment of TCO-doxorubicin at the tumor site after injection with tetrazine-modified sodium hyaluronate biopolymer, thereby exerting anti-cancer effects with low side effects. This is the first bioorthogonal reaction applied to patients (Wu et al., 2021). If the TCO is replaced with an isocyano group, the water solubility of the drug can be improved and the impact on the structure of the drug could be minimized due to its small size. More interestingly, such a functional pair is attractive in the labeling of proteins and carbohydrates. Certain strategies such as genetic incorporation and metabolic engineering have been developed to insert the isocyano group into these biomolecules, allowing direct imaging or modulation of these “recombinant” targets. While multicomponent reactions involving isocyanides and side chains of the target protein have been proposed to modify native proteins, it is not practical for living systems due to the requirement of multicomponent coordination. In addition to explore the reactivity of isocyanides in bioorthogonal chemistry, we are also looking forward to methods that can tag the endogenous targets in living systems with isocyano groups, to promote their wide applications.
