# Supplementary figures and images for: Isocyanides: Promising Functionalities in Bioorthogonal Labeling of Biomolecules
Source: Front Chem. 2021 Apr 29;9:670751. doi: 10.3389/fchem.2021.670751 (PMC8117350; doi:10.3389/fchem.2021.670751)

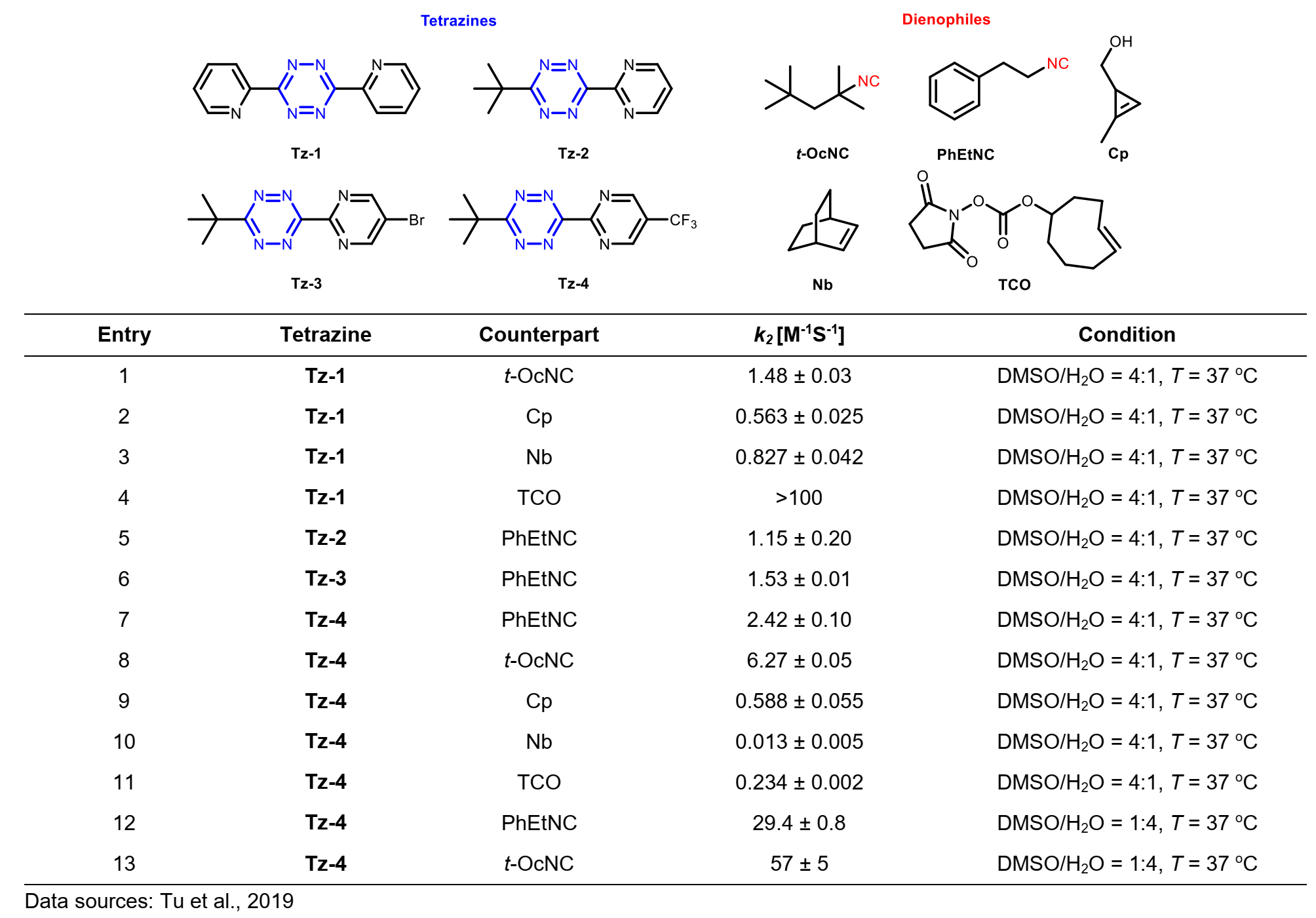

Supplement: Supplementary file 1 [file Image1.TIF]
